# *dnaJ*: a New Approach to Identify Species within the Genus Enterobacter

**DOI:** 10.1128/Spectrum.01242-21

**Published:** 2021-12-22

**Authors:** Enrique Hernandez-Alonso, Simon Barreault, Luis A. Augusto, Pierre Jatteau, Millie Villet, Pierre Tissieres, Florence Doucet-Populaire, Nadege Bourgeois-Nicolaos

**Affiliations:** a Université Paris-Saclay, CEA, CNRS, Institute for Integrative Biology of the Cell (I2BC), Gif-sur-Yvette, France; b Department of Bacteriology-Hygiene, AP-HP Université Paris-Saclay, Antoine Beclere Hospital, Clamart, France; c Department of Pediatrics Intensive Care and Neonatal Medicine, AP-HP Université Paris-Saclay, Bicêtre Hospital, Paris, France; d FHU Sepsis, AP-HP/Université Paris-Saclay/Inserm, Le Kremlin-Bicêtre, France; Memorial Sloan Kettering Cancer Center

**Keywords:** *Enterobacter bugandensis*, *Enterobacter cloacae*, *Enterobacter quasihormaechei*, *Enterobacter*, SeqScape software, *dnaJ* gene, gene PCR-sequencing, neonates, sepsis, species identification

## Abstract

The taxonomy of the genus Enterobacter can be confusing and has been considerably revised in recent years. We propose a PCR and amplicon sequencing technique based on a partial sequence of the *dnaJ* gene for species assignment consistent with DNA-DNA digital hybridization (dDDH) and pairwise average nucleotide identity (ANI). We performed a validation of the method by comparing the type strains of each species, sequences obtained from the GenBank database, and clinical specimens. Our results show that the polymorphism of the target sequence of *dnaJ* allows the identification of species. Using this gene, we assigned the species to 100 strains deposited in the GenBank database that were consistent with the species assignment by dDDH and ANI. The analysis showed that using the partial *dnaJ* sequence is congruent with WGS as far as correct identification of Enterobacter species is concerned. Finally, we applied our *dnaJ* method on a national collection of 68 strains identified as Enterobacter isolated from the blood cultures of premature babies using an algorithm based on a type-strain library and the SeqScape software. For the first time, we identified Enterobacter *quasihormaechei* in blood cultures from four neonatal sepsis cases. We also noticed a higher prevalence of *E. bugandensis* (36.3%; 32/88) and *E. xiangfangensis* (46.5%; 41/88). *E. bugandensis* is a novel species recently described specifically in instances of neonatal sepsis. In conclusion, sequencing a part of the *dnaJ* gene could be a quick, more economical, and highly discriminating method of identifying Enterobacter species in clinical practice and research.

**IMPORTANCE** We propose a new approach for Enterobacter species identification based on the diversity of the gene encoding the heat shock protein DnaJ. This new tool can be easily implemented in clinical laboratories in addition to identification by MALDI-TOF.

## INTRODUCTION

Enterobacter species are increasingly involved in human infections particularly among critically ill patients with sepsis. The incidence of these often-nosocomial infections is particularly high in neonatology where they are facilitated by immune incompetence linked to the immaturity of premature infants. (1, 2). The taxonomy of the genus Enterobacter has been reevaluated in the last 10 years. Some species have been reclassified into other genera such as Enterobacter aerogenes which has been moved to the genus Klebsiella (3). Recently, Wu et al. suggested that Enterobacter *timonensis* should have been moved to a new genus called *Pseudenterobacter* (4). Moreover, the concept of subspecies in the genus Enterobacter has been a source of confusion and is no longer recommended (4). In recent years the use of tools such as digital DNA-DNA hybridization (dDDH) and pairwise average nucleotide identity (ANI) have allowed a more accurate classification of this genus. In particular, the DNA-DNA hybridization (DDH) 70% cutoff is now regarded as the “gold standard” for species delineation (5). In practice, thresholds of 96.0% or 70.0% with ANI and dDDH (comparable with DDH for all intents and purposes) have been used, respectively, for species identification using Whole-Genome Sequencing (WGS) (6). The most recent taxonomic study of the genus Enterobacter according to the overall genome relatedness index (OGRI) using dDDH and ANI values between the type strains showed that *E. dissolvens*, *E. hoffmannii*, *E. hormaechei*, and *E. xiangfangensis* are independent species. Furthermore, *E. hormaechei* subsp. *oharae* and *E. hormaechei* subsp. *steigerwaltii* have been reclassified as *E. xiangfangensis* (4). Nowadays, the genus Enterobacter consists of 22 species (Table 1). In a recent study Wu et al. showed that *E. asburiae*, *E. bugandensis*, *E. cancerogenus*, *E. chengduensis,* E. cloacae, *E. hoffmannii*, *E. huaxiensis, E. kobei*, *E. ludwigii*, *E. quasiroggenkampii*, *E. roggenkampii,* and *E. xiangfangensis* are frequently isolated from blood cultures (4).

**TABLE 1:** Classification of the 22 type strains of the genus Enterobacter used in this study

Species (*n* = 22)	Accession	Type strain	Substitution rates^*a*^
Enterobacter asburiae	CP011863.1	JCM 6051	*S *= 183.3 *N *= 530.7 *d_S_* = 0.2624 *d_N_* = 0.0039 *d_N_*/*d_S_* = 0.0147
Enterobacter bugandensis	LT992502.1	EB-247	*S *= 187.8 *N *= 526.2 *d_S_* = 0.2265 *d_N_* = 0.0019 *d_N_*/*d_S_* = 0.0086
Enterobacter cancerogenus	ERR1854846	ATCC 35316	*S *= 190.2 *N *= 523.8 *d_S_* = 0.3061 *d_N_* = 0.0098 *d_N_*/*d_S_* = 0.0322
Enterobacter *chengduensis*	CP043318.1	WCHECI-C4	*S *= 175.8 *N *= 538.2 *d_S_* = 0.2814 *d_N_* = 0.0038 *d_N_*/*d_S_* = 0.0136
Enterobacter *chuandaensis*	GCF_003594915.1	090028	*S *= 213.6 *N *= 500.4 *d_S_* = 0.2007 *d_N_* = 0.0021 *d_N_*/*d_S_* = 0.0103
Enterobacter cloacae	CP001918.1	ATCC 13047	*S *= 186.7 *N *= 527.3 *d_S_* = 0.3218 *d_N_* = 0.0019 *d_N_*/*d_S_* = 0.0060
Enterobacter dissolvens	WJWQ01000001.1	ATCC 23373	*S *= 192.5 *N *= 521.5 *d_S_* = 0.3190 *d_N_* = 0.0020 *d_N_*/*d_S_* = 0.0061
Enterobacter *hoffmannii*	CP017186.1	DSM 14563	*S *= 180.2 *N *= 533.8 *d_S_* = 0.2423 *d_N_* = 0.0019 *d_N_*/*d_S_* = 0.0079
Enterobacter hormaechei	MKEQ01000001.1	ATCC 49162	*S *= 182.6 *N *= 531.4 *d_S_* = 0.2536 *d_N_* = 0.0019 *d_N_*/*d_S_* = 0.0076
Enterobacter *huaxiensis*	QZCT01000001.1	090008	*S *= 181.6 *N *= 532.4 *d_S_* = 0.3211 *d_N_* = 0.0097 *d_N_*/*d_S_* = 0.0301
Enterobacter kobei	CP017181.1	ATCC BAA-260	*S *= 189.0 *N *= 525.0 *d_S_* = 0.2929 *d_N_* = 0.0019 *d_N_*/*d_S_* = 0.0066
Enterobacter ludwigii	CP017279.1	EN-119	*S *= 185.6 *N *= 528.4 *d_S_* = 0.3056 *d_N_* = 0.0039 *d_N_*/*d_S_* = 0.0126
Enterobacter mori	AEXB00000000.1	LMG 25706	*S *= 189.0 *N *= 525.0 *d_S_* = 0.1990 *d_N_* = 0.0039 *d_N_*/*d_S_* = 0.0198
Enterobacter *oligotrophica*	AP019007.1	CCA6	*S *= 183.8 *N *= 530.2 *d_S_* = 0.2296 *d_N_* = 0.0039 *d_N_*/*d_S_* = 0.0169
Enterobacter *quasihormaechei*	SJON01000001.1	WCHEs120003	*S *= 181.4 *N *= 532.6 *d_S_* = 0.2401 *d_N_* = 0.0058 *d_N_*/*d_S_* = 0.0242
Enterobacter *quasimori*	RXRX00000000.1	090044	*S *= 181.1 *N *= 532.9 *d_S_* = 0.2380 *d_N_* = 0.0019 *d_N_*/*d_S_* = 0.0081
Enterobacter *quasiroggenkampii*	LFDQ00000000.2	WCHECL1060	*S *= 191.5 *N *= 522.5 *d_S_* = 0.2672 *d_N_* = 0.0020 *d_N_*/*d_S_* = 0.0074
Enterobacter *roggenkampii*	CP017184.1	DSM 16690	*S *= 191.4 *N *= 522.6 *d_S_* = 0.2424 *d_N_* = 0.0020 *d_N_*/*d_S_* = 0.0081
Enterobacter *sichuanensis*	POVL01000001.1	WCHECI1597	*S *= 194.7 *N *= 519.3 *d_S_* = 0.3025 *d_N_* = 0.0020 *d_N_*/*d_S_* = 0.0065
Enterobacter soli	LXES01000001.1	ATCC BAA-2102	*S *= 202.9 *N *= 511.1 *d_S_* = 0.4126 *d_N_* = 0.0101 *d_N_*/*d_S_* = 0.0244
Enterobacter *wuhouensis*	S JOO01000001.1	WCHES120002	*S *= 187.3 *N *= 526.7 *d_S_* = 0.2919 *d_N_* = 0.0059 *d_N_*/*d_S_* = 0.0201
Enterobacter xiangfangensis	CP017183.1	LMG 27195	*S *= 191.6 *N *= 522.4 *d_S_* = 0.1984 *d_N_* = 0.0020 *d_N_*/*d_S_* = 0.0099

^*a*^The parameters were calculated using the web server PAL2NAL (18). *S*, number of synonymous sites; *N*, number of nonsynonymous sites; *d_s_*, synonymous substitution rate; *d_N_*, nonsynonymous substitution rate.

Correct species identification within the genus Enterobacter is still a challenge. Matrix-assisted laser desorption ionization–time of flight mass spectrum (MALDI-TOF MS) is the major tool used in clinical laboratories for bacterial identification. However, species identification using MALDI-TOF MS may have its limits (7). In clinical laboratories, tests based on the phenotype and the MALDI-TOF MS usually identify Enterobacter isolates as E. cloacae, thereby neglecting the clinical importance of other species in the hospital environment (8). Nevertheless, a correct identification on the species-level is crucial for epidemiology, pathogenesis, diagnosis, treatment, prognosis, and prevention (9, 10). According to Wu et al.’s precise species study, among the 1,960 Enterobacter strains submitted in the GenBank, only 80 (14.8%) of the 540 genomes labeled E. cloacae actually belonged to these species (4). Several other species of the genus Enterobacter have also been associated with infections, particularly *E. bugandensis* in neonatal sepsis (11).

Although the WGS allows correct identification on the species-level, this tool is still far from routine in clinical practice. It requires highly trained personnel for data assembly and analysis, in addition to being too expensive to use in many countries. Faster and more affordable methods of the species identification correlated with WGS as regards to species identification, are essential in clinical microbiology.

The *dnaJ* housekeeping gene that encodes the heat shock protein 40 (Hsp40) has been shown to have a high discriminating power in *Enterobacterales* showing better resolution than 16S *r*RNA, *tuf, hsp60, or atpD* (12, 13). The *dnaJ* gene contains an immutable sequence within each species of the genus Enterobacter that can be used for accurate identification (14, 15).

In this study, we propose a PCR and amplicon sequencing method to correctly identify species within the genus Enterobacter using a partial sequence of the *dnaJ* gene. Our method provides a highly discriminating tool in clinical practice.

## RESULTS

### *dnaJ* polymorphism analysis in Enterobacter type strains.

**FIG 1:**
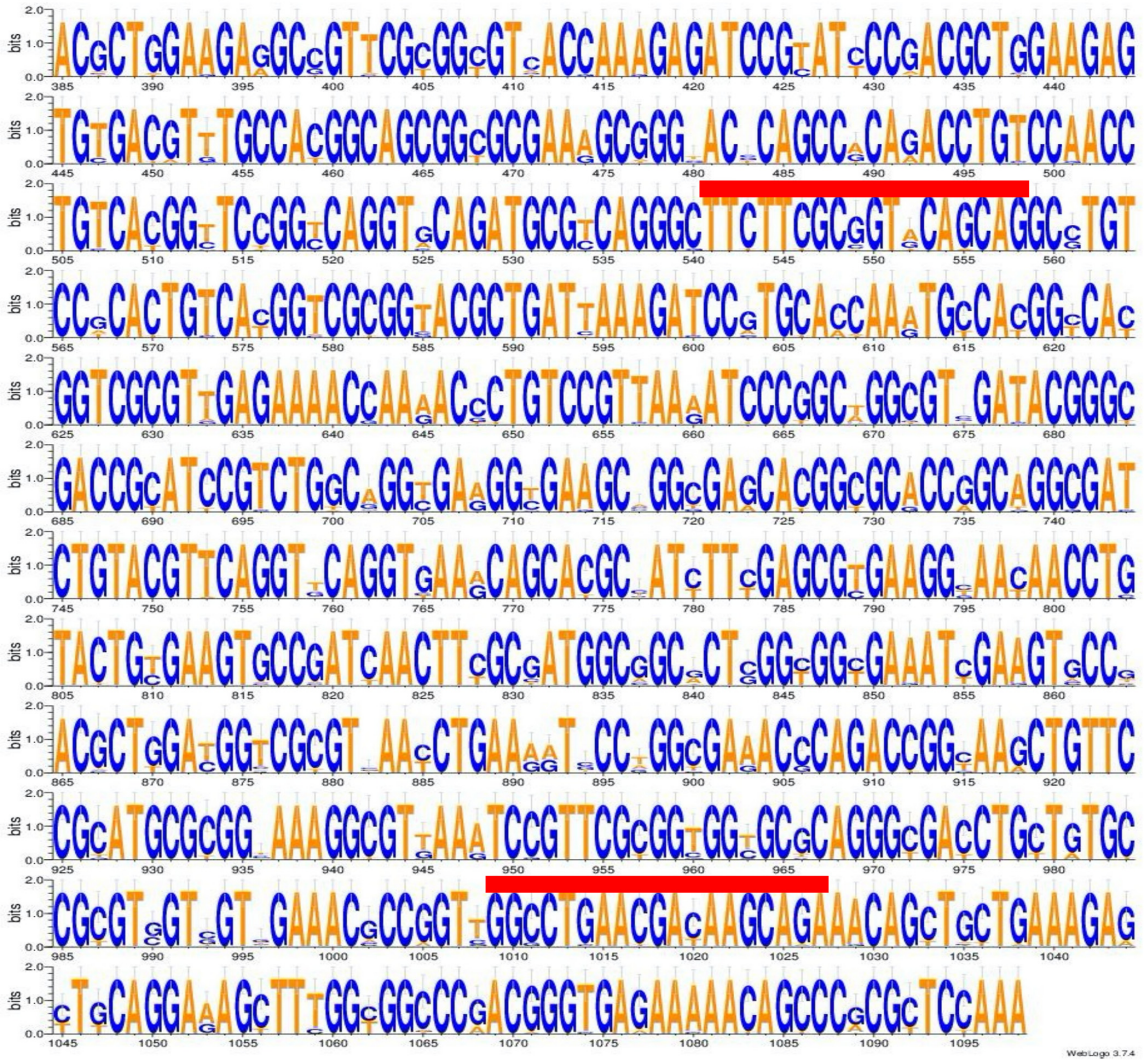
Logo plot of partial sequence of the *dnaJ* gene from position 385 to 1098 (714 bp). Sequence sets are shown relative to a *dnaJ* sequence of 22 different type strains of the genus Enterobacter (Table S2). The DNA fragment is characterized by the presence of two regions highly variable but conserved intraspecies (positions 541 to 558 and positions 1009 to 1027, showed by red line) descripted previously (14). All DNA sequences are listed 5′ to 3′.

**FIG 2:**
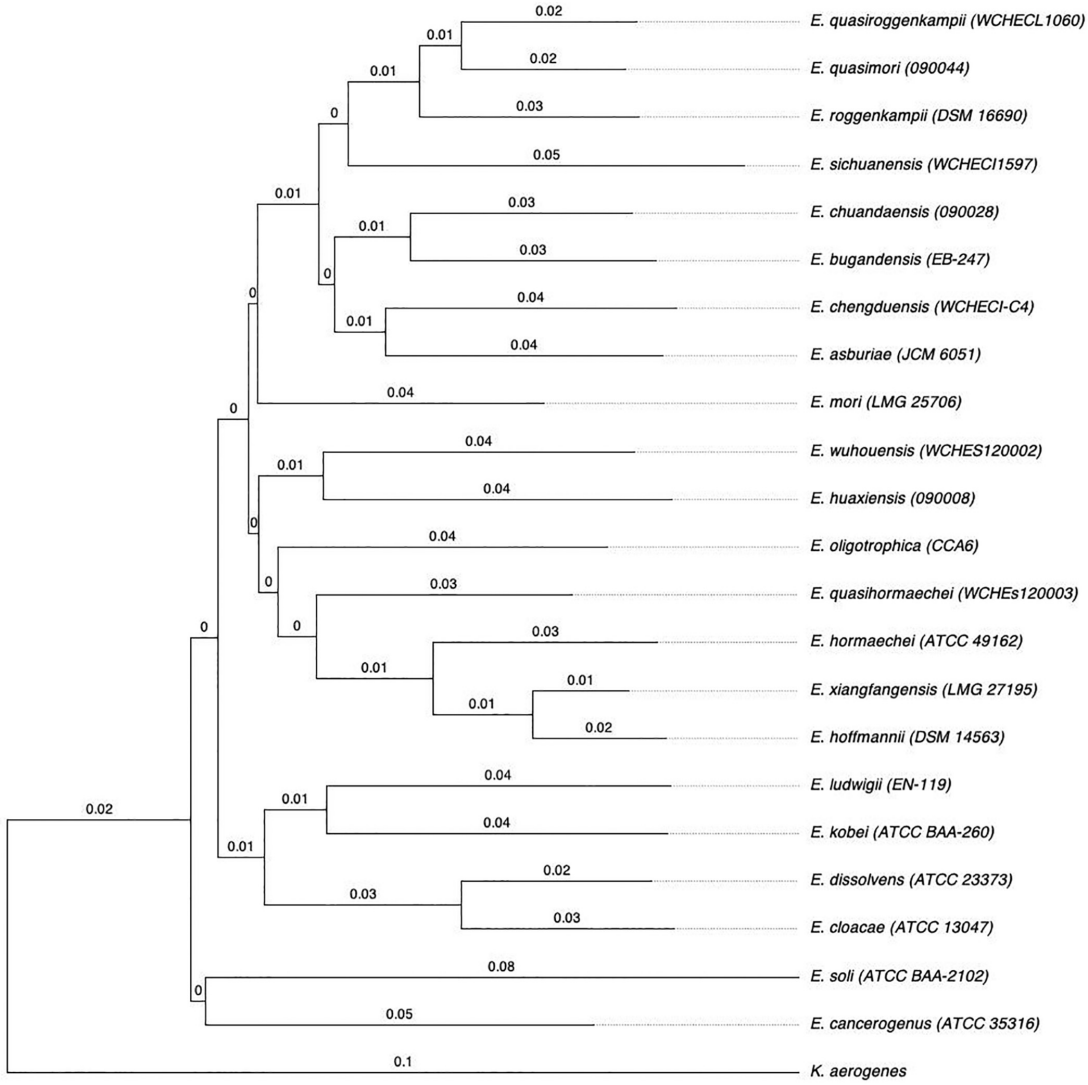
Molecular phylogenetic analysis of the partial sequence of the *dnaJ* gene of the type strains. The tree was inferred using the Neighbor-Joining method under the Tamura–Nei model with a 1000-bootstrap test. The analysis involved 22 partial nucleotide sequences (714 bp) of the *dnaJ* gene. The tree was rooted with the *dnaJ* sequence of K. aerogenes download from GenBank (AB008141.1). The *dnaJ* gene partial sequences of the type strains used are available in the Table S2.

The genomes of the 22 type strains were obtained from GenBank (Table 1). We extracted the partial sequence of the *dnaJ* gene from position 385 to 1098 (714 bp), a region previously described for the analysis of species in *Enterobacterales* (14). The DNA fragment is characterized by the presence of two highly variable regions but identical within the same species (positions 541 to 558 and positions 1009 to 1027). We performed the alignment of the 22 sequences of the type strains. The polymorphism within the sequence was visually inspected (Fig. 1). Visual analysis showed a variation from 3.8% to 19.3% (corresponding to 27–138 different nucleotides). Using the alignment of the partial sequences of the *dnaJ* gene of the 22 type strains, we identified a percentage of similarity ranging from 87.8% to 95.9% (Table S1). The phylogenetic tree based on the target sequence of the *dnaJ* gene showed that each species of the genus forms an independent branch separated from the others that allows for species discrimination (Fig. 2). In addition, we determined the synonymous and nonsynonymous substitutions for a better understanding of the evolutive molecular dynamics of the sequence (Table 1). In all type strains, the *d_N_/d_S_* ratio was well below 1, clearly indicating a negative selection.

### *In silico* validation of species-level identification using *dnaJ.*

**FIG 3:**
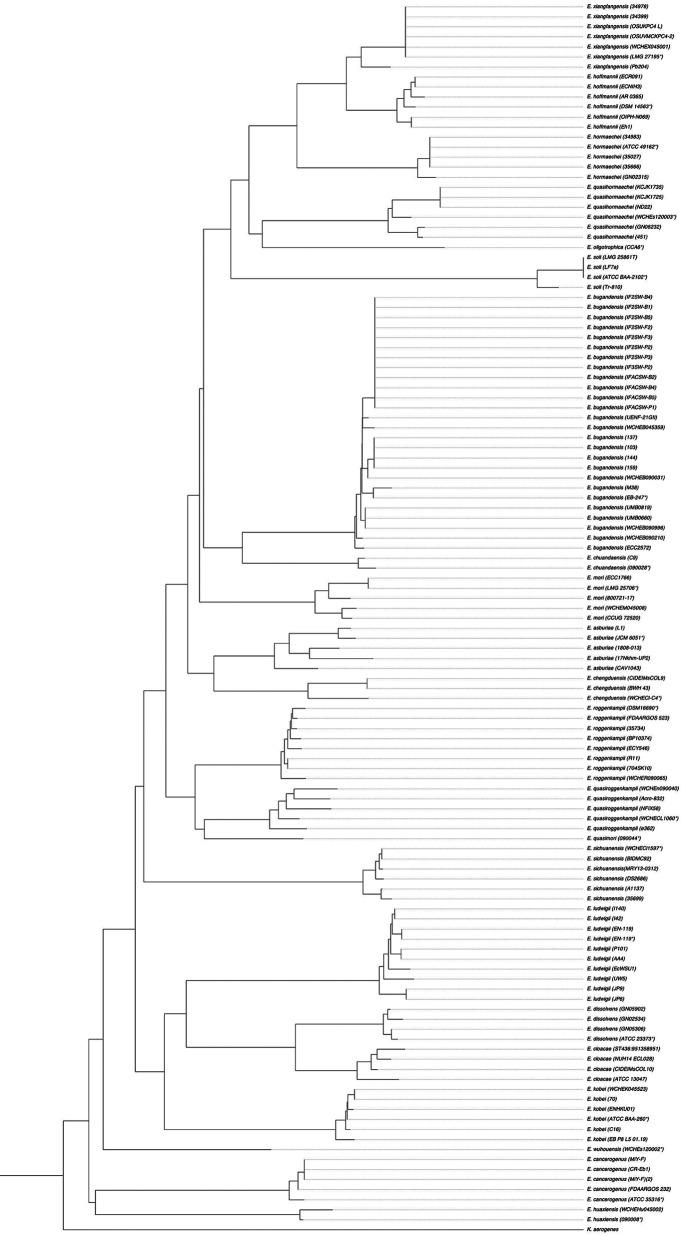
Molecular phylogenetic analysis of the partial sequence of the *dnaJ* gene between GenBank strains with the type strains (*). The tree was inferred using the Neighbor-Joining method under the Tamura–Nei model with a 1000-bootstrap test. The analysis involved 122 partial nucleotide sequences (714 bp) of the *dnaJ* gene. The tree was rooted with the *dnaJ* sequence of K. aerogenes download from GenBank (AB008141.1). All 122 *dnaJ* gene partial sequences are available in the Table S2.

We downloaded the genome of the 100 Enterobacter strains of interest that were taxonomically corrected in 2020 to determine the relationship between their genomes and that of the type strains by dDDH (Table S2). We found an interval between 74.4% and 100% as did other studies (3). Next, we extracted the 714 bp partial sequence of the *dnaJ* gene from those 100 strains and established the phylogenetic relationship in conjunction with the 22 *dnaJ* partial sequences of the type strains. The resulting phylogenetic tree showed separate branches for each species as was the case with dDDH (Fig. 3).

### Species-identification using PCR and amplification sequencing of *dnaJ* is consistent with the results of the ANI and dDDH methods.

**FIG 4:**
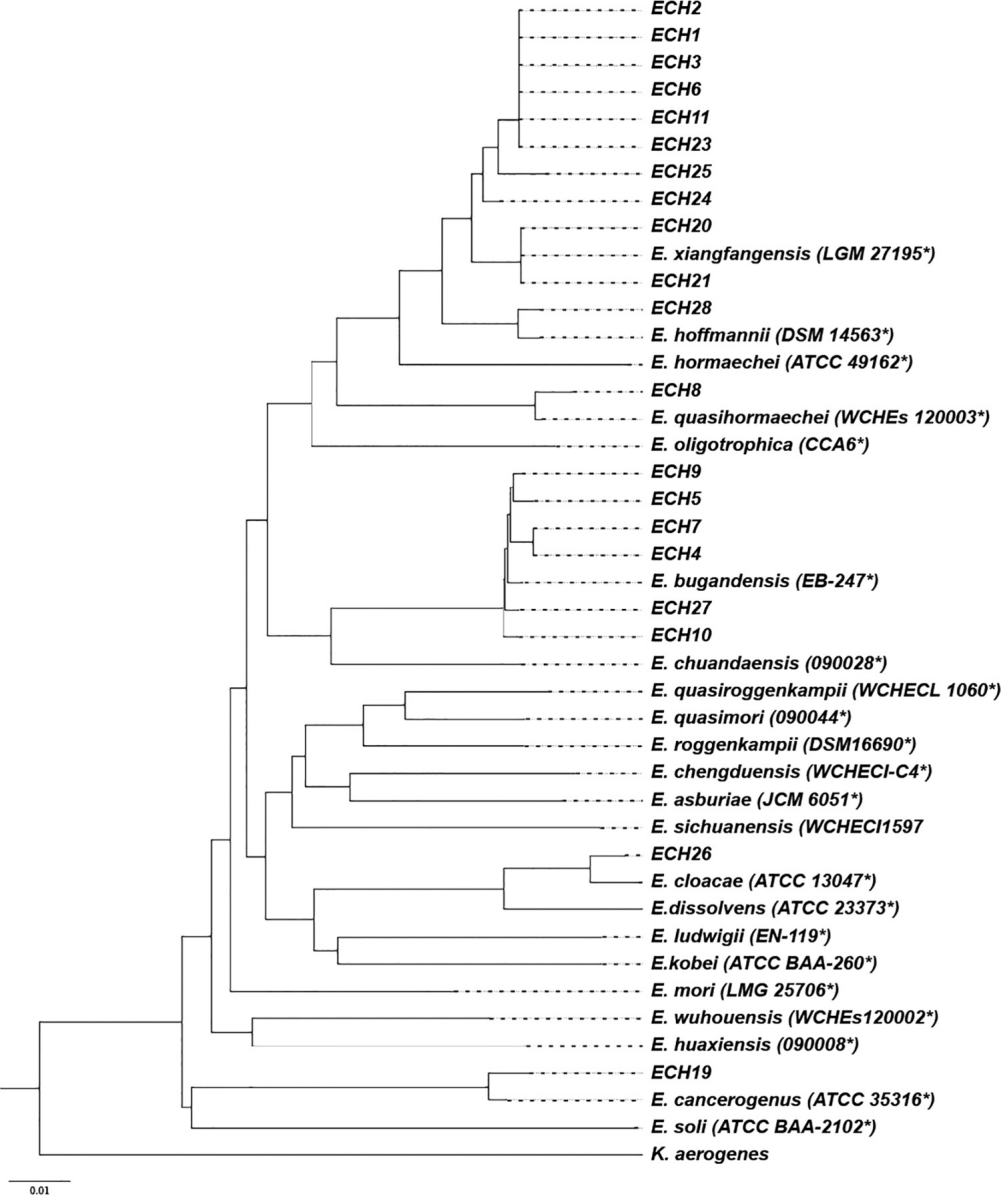
Molecular phylogenetic analysis of the partial sequence of the *dnaJ* gene of type strains and 20 relevant clinical strains from blood culture sequenced by WGS. The tree was inferred using the Neighbor-Joining method under the Tamura–Nei model with a 1000-bootstrap test. The analysis involved 42 partial nucleotide sequences (714 bp) of the *dnaJ* gene. The tree was rooted with the *dnaJ* sequence of K. aerogenes download from GenBank (AB008141.1). The *dnaJ* gene partial sequences of the type strains (*) used are available in table Supplementary text file 1 and the 20 clinical strains sequences are available in GenBank with the access number from MT665008.1 to MT665027.1.

First, we used the same 20 Enterobacter clinical strains previously sequenced by WGS in our laboratory (BioProject access number: PRJNA770343) in order to perform the PCR and amplicon sequencing of the *dnaJ* gene. After amplification, the 714 bp amplicon of each strain was sequenced, and the alignment and construction of the phylogenetic tree was carried out in conjunction with the type strains. The tree shows a clear distribution of the different species based on the sequence of the *dnaJ* gene (Fig. 4). According to the results of the phylogenetic analysis, the species were assigned as *E. bugandensis, E. cancerogenus*, E. cloacae, *E. hoffmannii*, *E. quasihormaechei*, and *E. xiangfangensis* (Table 2).

**TABLE 2:** Comparison of species identification of Enterobacter clinical strains by MALDI-TOF MS and *dnaJ* gene sequencing as well as OGRI

Strain	MALDI-TOF MS				*dnaJ*		OGRI	
	Organism identified	Score			Organism identified^*a*^		ANI	dDDH
ECH1	E. cloacae	2.12			Enterobacter xiangfangensis		99.00	91.70
ECH2	E. cloacae	2.09			Enterobacter xiangfangensis		98.98	91.00
ECH3	E. cloacae	2.08			Enterobacter xiangfangensis		99.01	91.60
ECH4	E. cloacae	2.19			Enterobacter bugandensis		98.69	92.70
ECH5	*E. asburiae*	2.00			Enterobacter bugandensis		98.53	95.80
ECH6	E. cloacae	2.23			Enterobacter xiangfangensis		99.99	91.60
ECH7	E. cloacae	2.05			Enterobacter bugandensis		98.68	93.80
ECH8	E. cloacae	2.14			Enterobacter *quasihormaechei*		98.76	95.80
ECH9	*E. asburiae*	2.02			Enterobacter bugandensis		98.70	95.40
ECH10	*E. asburiae*	2.11			Enterobacter bugandensis		98.37	96.80
ECH11	E. cloacae	2.12			Enterobacter xiangfangensis		99.00	91.40
ECH19	*E. cancerogenus*	2.12			Enterobacter cancerogenus		99.14	91.30
ECH20	E. cloacae	2.24			Enterobacter xiangfangensis		99.26	93.90
ECH21	E. cloacae	2.21			Enterobacter xiangfangensis		99.24	94.00
ECH23	E. cloacae	2.16			Enterobacter xiangfangensis		98.99	91.20
ECH24	E. cloacae	2.18			Enterobacter xiangfangensis		99.03	91.70
ECH25	E. cloacae	2.18			Enterobacter xiangfangensis		99.19	93.50
ECH26	E. cloacae	2.09			Enterobacter cloacae		98.68	88.90
ECH27	*E. kobei*	2.06			Enterobacter bugandensis		98.44	87.00
ECH28	E. cloacae	2.16			Enterobacter *hoffmannii*		98.44	93.30

^*a*^Species assignation according to the most recent classification. OGRI, overall genome relatedness index; ANI, average nucleotide identity; dDDH, digital DNA-DNA hybridization.

Second, we used the WGS data to confirm the species using ANI and dDDH values. We identified a range from 98.4% to 99.9% and 87.0% to 96.8% for ANI and dDDH, respectively (Table 2). The results showed a correct species assignment using *dnaJ* confirmed by the ANI and dDDH methods for 100% of the strains studied.

### Application of the *dnaJ* target sequencing to clinical specimens.

**FIG 5:**
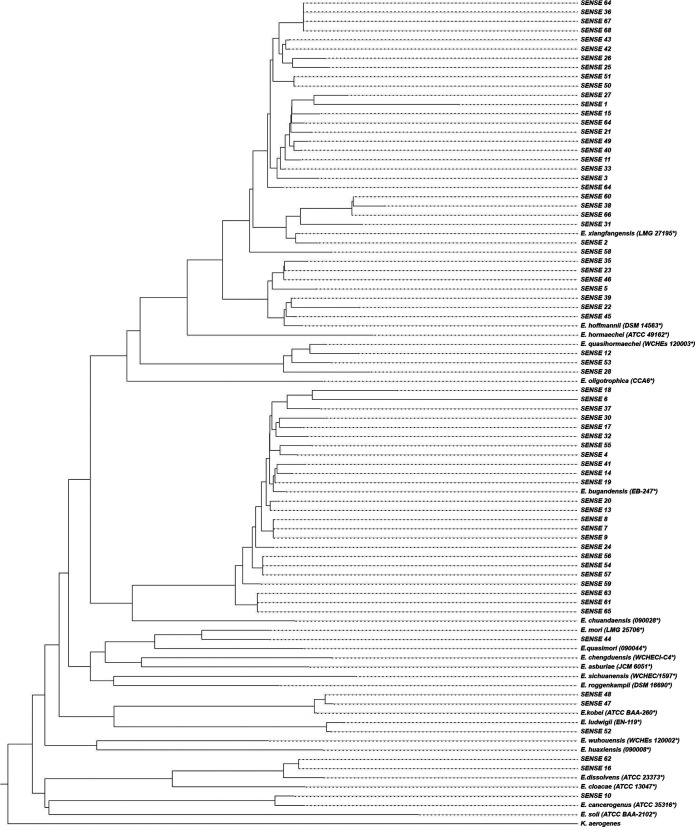
Molecular phylogenetic analysis of the partial sequence of the *dnaJ* gene between SENSE strains with the type strains (*). The tree was inferred using the Neighbor-Joining method under the Tamura–Nei model with a 1000-bootstrap test. The analysis involved 68 partial nucleotide sequences (714 bp) of the *dnaJ* gene. The tree was rooted with the *dnaJ* sequence of K. aerogenes download from GenBank (AB008141.1).

First, to verify the usefulness of this new technique based on the PCR and amplicon sequencing of the target *dnaJ* sequence as a diagnostic tool, we retrospectively analyzed 68 blood cultures isolates (named SENSE1-68) previously identified using MALDI-TOF MS as E. cloacae complex. We found 9 different species: *E. bugandensis* (*n = 24*), *E. cancerogenus* (*n = 1*), *E. dissolvens* (*n = 2*), *E. hoffmannii* (*n = 7*), *E. kobei* (*n = 2*), *E. ludwigii* (*n = 1*), *E. mori* (*n = 1*), *E. quasihormaechei* (*n = 3*), and *E. xiangfangensis* (*n = 27*). The most prevalent was *E. xiangfangensis* (40.0%) followed by *E. bugandensis* (35.0%) (Fig. 5).

Second, we developed a local library using all the 22 partial sequences of *dnaJ* of the type strains in order to implement this method in the microbiology department of our hospital. This library was uploaded in the SeqScape software that provides library functions for comparison to a known group of sequences. After Sanger sequencing of partial sequence of *dnaJ* of 68 Enterobacter clinical strains from a national collection (16) all sequencing files were compared with our library. All the 68 strains (100.0%) matched with the species previously identified by phylogenetic analysis.

## DISCUSSION

The correct identification of bacterial isolates is crucial to patient care. It is essential in predicting the clinical prognosis as well as in choosing an adapted antibiotic regimen. However, for the genus Enterobacter, species identification is complicated. The traditional methods used in clinical laboratories, fail to correctly identify the species. Therefore, other methods are needed. Pavlovic et al. observed that MALDI-TOF MS was inadequate to differentiate *E. asburiae*, *E. hormaechei*, *E. kobei,* and *E. ludwigii* from E. cloacae (15). We developed a method based on a partial sequence of the *dnaJ* gene for species identification within the genus Enterobacter.

*In silico* analysis of the partial sequence of the *dnaJ* gene of the type strains showed a high interspecies polymorphism verified by the analysis of the phylogenetic evolution of the partial sequence of the genus Enterobacter (*d_N_/d_S_* <1). We observed that the polymorphism of the *dnaJ* target sequence allows precise Enterobacter species identification.

As dDDH values have often been used in taxonomic studies, 100 recent genomes of different Enterobacter species were compared with the type strains to verify the correct species assignment. We observed a range from 74.4% to 100.0%. Our results were consistent with the Wu et al. study (4). We used these genome data to extract the partial sequence of the *dnaJ* gene for phylogenetic analysis and species assignment. The results show 100% accurate species assignment of the strains analyzed using *dnaJ* verified by dDDH.

A phylogenetic analysis based on the *dnaJ* gene supports the idea that, using only this partial sequence, it is possible to obtain correct species identification in the genus Enterobacter (Fig. 2 and 3). The method described here allows for a correct and rapid species identification as valid as OGRI.

Using our PCR-sequencing *dnaJ* method, we observed that 18/20 (86.0%) of the whole-genome sequenced Enterobacter clinical strains were misidentified by MALDI-TOF MS. Only one strain among 15 (6.0%) corresponded to E. cloacae. 3 *E. asburiae* and 1 *E. kobei* were identified as *E. bugandensis*,11 *E. xiangfangensis* were misidentified as E. cloacae, and one E. cloacae was *identified* as *E. hoffmannii* (Table 2). Those results were correlated with OGRI analysis and dDDH.

Then we applied our method on a historical sampling of 68 blood culture Enterobacter isolates from neonates. We found a high prevalence (40.0%) of *E. xiangfangensis*. We also identified *E. quasihormaechei,* a novel species reported in 2020 (17). *E. quasihormaechei* could share the same virulence and pathogenicity mechanisms as *E. xiangfangensis,* previously identified in other studies as *E. hormaechei,* a species that often exhibits resistance to multiple commonly used antibiotics and persists in nosocomial environments (18). In this study, we report for the first time to our knowledge, four *E. quasihormaechei* isolates from blood cultures in patients with neonatal sepsis.

We also detected a high prevalence of *E. bugandensis*, another novel enterobacterial species associated with severe clinical infections, particularly with neonatal sepsis, suggesting that this element of the genus Enterobacter is an emergent species in Europe (19, 20). *E. bugandensis* is considered to be a highly pathogenic Enterobacter species with specific virulence mechanisms and usually a multidrug resistant profile (7, 10). Our preliminary analysis showed that our isolates identified as *E. bugandensis* are susceptible to third generation cephalosporins revealing an antibiotic resistance profile different from that previously reported (data not shown) (19).

Several epidemiological studies reported a high prevalence of E. cloacae in clinical isolates which is contrary to our findings where E. cloacae is less prevalent. The high prevalence of E. cloacae in other studies could be explained by species misidentification in the genus Enterobacter (21–23). In clinical practice bacterial identification is necessary for a better understanding of pathogenesis, virulence, and resistance to antibiotics. The fact is that identifying Enterobacter species has considerable medical significance since each species behaves differently: for instance, some are more likely to lead to nosocomial outbreaks while other are more prone to developpe resistance to antibiotics. For example, in a recent study, we showed that *E. bugandensis* is more virulent in neonates than other species due to the presence of specific factors of virulence (24). Moreover, *E. xiangfangensis* and *E. steigerwaltii* have the highest prevalence of resistance of third generation cephalosporins and these two species were found to be especially prone to produce carbapenemases (25). In the future, the correct identification of species of the genus Enterobacter will also improve the understanding of their epidemiology in the clinical environment and will permit implemention of better strategies in the prevention of health care-associated infections. It will also improve patients’ prognosis and treatment.

In conclusion, our method, based on a partial *dnaJ* gene PCR and amplicon sequencing, could be used in clinical practice as a specific, rapid, and highly discriminating tool, with similar results to those obtained using OGRI methods, for the correct identification of Enterobacter species. This improvement in correct identification can also facilitate the management of nosocomial outbreaks and promote rapid environmental monitoring.

## MATERIALS AND METHODS

### Phylogenetic and *in silico* analysis of the partial sequence of the *dnaJ* gene in type strains.

The 22 type strain genomes were downloaded from GenBank for *in silico* analysis. We aligned the *dnaJ* gene (1146 bp) and then extracted a partial sequence from position 385 to 1098 (714 bp). The alignment and pairwise distancing were performed using the MUSCLE method (26). The Neighbor-Joining phylogenetic tree was constructed with the Tamura–Nei model and a 1000-bootstrap test using MegAlign Pro (DNASTAR) and MEGAX (27). We determined the numbers of synonymous (S) and nonsynonymous (N) sites as well as the rates of synonymous (*d*_S_) and nonsynonymous (*d*_N_) substitutions using the web site http://abacus.gene.ucl.ac.uk/software/paml.html (28).

### Determination of overall genome relatedness.

The dDDH among 100 genomes selected from GenBank (Table S1) and type strains of Enterobacter species were determined using the web-service http://ggdc.dsmz.de (formula 2). The pairwise ANI and dDDH among 20 clinically relevant bacterial isolates (Table 2) and type strains of Enterobacter species were determined using the web-service http://enve-omics.ce.gatech.edu/ani/ for ANI and the web-service GGDC for dDDH. A ≥70% dDDH or a ≥96% ANI value was used as the cutoff to species assignation (29). The genome of the 20 clinically relevant strains conveniently used in this study have been deposited at DDBJ/ENA/GenBank under the BioProject accession number PRJNA770343.

### Designing of primers, PCR, and amplicon sequencing of the partial *dnaJ* gene.

Degenerate primers Hsp40-Fw (5′-GACCTGCGCTACAACATGGAKCT-3′) and Hsp40-Rv (5′-CCGCGYTCCAAAAGCTTCTTYGAT-3′) were visually designed and analyzed (OligoAnalyzer) according to the alignment of the *dnaJ* gene sequences of the type strains. The primers were used to amplify a fragment of 750 bp. The amplification reaction was performed with Master Mix GoTaq (PROMEGA, USA), with 0.5 µl of each primer (0.2 µM final PCR concentration), and 2.5 µl of DNA template in a final reaction volume of 25 µl. PCR amplification was carried out in a thermal cycler (VERITI, Applied Biosystems) as follows: 4 min denaturation step at 94°C, followed by 30 cycles at 94°C for 50 s, 60°C for 35 s, and 72°C for 1 min, with a final extension step of 5 min at 72°C. PCR products were verified by agarose gel electrophoresis, purified using WizardSV gel and PCR clean Up System (Promega), and then sequenced using the Sanger sequencing technology with Hsp40-Fw/Hsp40-Rv primers by BigDye Terminator Cycle Sequencing Ready Reaction kit (Applied Biosystems, USA) according to the manufacturer’s instructions. After sequencing, primer sequences were removed and partial sequences of the *dnaJ* gene (714 bp) were confirmed by at least two chromatograms (forward and reverse) for the phylogenetic analysis.

### Bacterial strains.

Eighty-eight Enterobacter spp. strains isolated from blood cultures of neonate patients with sepsis were included. The strains were divided in 2 groups. The first 20 strains, already sequenced by WGS were used, to validate our method (BioProject access number: PRJNA770343) (24).

The second group included 60-eight Enterobacter spp. clinical strains collected from 8 French neonatal intensive care units (NICUs) known as the SENSE group between 2016 to 2019. They were grown on tryptic soy agar at 37°C for 24 h in aerobic conditions, and all isolates were identified twice as E. cloacae complex in the respective hospitals and in our laboratory by MALDI-TOF MS (Bruker, Leipzig, Germany). Microbial suspension at 1 on the McFarland scale was employed and 200 µl were used for DNA extraction following the easyMAG protocol (bioMérieux, France) according to the manufacturer’s instructions.

### Species identification in clinical practice.

We developed a local library using the partial sequence of *dnaJ*. This library allows comparison of the *dnaJ* sequence of clinical isolates with the sequences of the type strains. The local library was uploaded in the SeqScape software v4.0 (Applied Biosystems, Courtaboeuf, France). The analyze protocol was generated according to the manufactured recommendations. After Sanger sequencing, the amplicon sequences were analyzed with SeqSacape Software v4.0. We employed original sequencing files and corrected sequencing files. The species identification of a submitted clinical isolate sequence was given when the software algorithm assigns to each possible way of arrangement a score, which is defined as the standard measure of sequence similarity within a set of aligned sequences (30). A score of 800 was considered a cutoff value corresponding to 98.0–100.0% of homology between type strains and clinical isolates (Fig. S1).

### Data availability.

Sequence data generated by this study is available at BioProject PRJNA770343 (Whole Genome Shotgun project has been deposited at DDBJ/ENA/GenBank. The version described in this paper is version JAJAPC000000000-JAJAPV000000000).

The GenBank accession numbers for the sequences reported in this paper for the *dnaJ* gene of these 20 Enterobacter isolates range from MT665008.1 to MT665027.1.
