# Supplementary material for: dnaJ: a New Approach to Identify Species within the Genus Enterobacter
Source: Microbiol Spectr. 2021 Dec 22;9(3):e01242-21. doi: 10.1128/Spectrum.01242-21 (PMC8694106; doi:10.1128/Spectrum.01242-21)
Supplement: SUPPLEMENTAL FILE 1 — Supplemental material. Download SPECTRUM01242-21_Supp_1_seq11.pdf, PDF file, 0.4 MB [file spectrum01242-21_supp_1_seq11.pdf]

Description of Supplementary Files

File name: **Table S1. Homology percentage between the type strains within the genus *Enterobacter*.**

Fie name: **FIG S1. Library report using SeqScape® Software v.4.0.** S13 strain was identified as *E. bugandensis* with a score of 950 and 0 mismatches. The software shows all mismatches and respective score by comparison with all type strains in the library.

Table S1. Homology percentage between the type strains within the genus *Enterobacter*.

	**1**	**2**	**3**	**4**	**5**	**6**	**7**	**8**	**9**	**10**	**11**	**12**	**13**	**14**	**15**	**16**	**17**	**18**	**19**	**20**	**21**	**22**
**1**	90.66	90.10	100.00	93.03	93.03	92.19	89.12	92.47	92.19	92.19	91.49	91.07	90.38	92.47	92.19	92.33	90.79	91.35	91.35	91.21	93.58	90.52
**2**	90.24	89.96	93.03	100.00	94.56	91.91	89.96	93.03	92.33	92.47	92.33	92.33	90.38	93.31	92.89	93.03	90.66	89.96	91.21	90.52	92.89	91.35
**3**	100.00	90.52	90.66	90.24	89.96	91.63	89.68	91.49	90.52	90.93	90.38	90.38	91.63	90.52	90.93	90.93	90.38	91.35	91.35	89.96	89.68	90.79
**4**	89.68	89.96	93.58	92.89	92.61	91.77	89.12	93.17	91.35	91.77	91.07	91.35	90.79	94.00	93.58	93.31	89.96	90.38	91.21	91.91	100.00	90.52
**5**	89.96	90.93	93.03	94.56	100.00	91.91	88.84	93.17	92.33	91.91	92.47	92.47	91.07	93.72	93.58	93.73	90.10	90.38	91.49	91.21	92.61	91.35
**6**	90.38	91.49	90.79	90.66	90.10	91.35	89.54	91.77	89.82	90.38	90.79	90.52	89.96	90.79	90.38	90.79	100.00	95.26	90.52	90.66	89.96	90.93
**7**	91.35	91.77	91.35	89.96	90.38	91.21	89.26	91.77	90.52	90.24	90.24	89.82	89.40	91.63	90.93	91.21	95.26	100.00	90.38	91.21	90.38	91.07
**8**	90.38	90.52	91.49	92.33	92.47	92.75	88.84	91.91	94.98	93.58	100.00	97.21	90.66	90.79	91.49	91.63	90.79	90.24	91.35	90.24	91.07	90.38
**9**	90.52	91.21	92.19	92.33	92.33	91.77	89.96	91.77	100.00	92.75	94.98	94.98	91.21	91.07	91.21	91.49	89.82	90.52	90.38	90.66	91.35	90.93
**10**	91.63	90.24	90.38	90.38	91.07	92.47	88.84	92.47	91.21	92.33	90.66	91.63	100.00	89.96	91.21	90.10	89.96	89.40	92.75	90.10	90.79	89.26
**11**	90.79	92.47	90.52	91.35	91.35	91.21	88.70	90.93	90.93	90.38	90.38	90.66	89.26	91.91	92.19	92.33	90.93	91.07	91.77	90.66	90.52	100.00
**12**	90.52	100.00	90.10	89.96	90.93	91.77	88.28	91.49	91.21	91.35	90.52	90.79	90.24	91.07	90.24	91.21	91.49	91.77	91.49	90.66	89.96	92.47
**13**	91.49	91.49	92.47	93.03	93.17	92.47	89.54	100.00	91.77	94.14	91.91	92.33	92.47	92.33	94.84	92.61	91.77	91.77	92.47	90.10	93.17	90.93
**14**	91.63	91.77	92.19	91.91	91.91	100.00	89.40	92.47	91.77	92.89	92.75	93.17	92.47	92.33	91.77	92.19	91.35	91.21	91.49	90.66	91.77	91.21
**15**	90.93	91.35	92.19	92.47	91.91	92.89	89.68	94.14	92.75	100.00	93.58	94.28	92.33	91.63	92.61	90.93	90.38	90.24	92.33	90.52	91.77	90.38
**16**	90.93	90.24	92.19	92.89	93.58	91.77	87.87	94.84	91.21	92.61	91.49	91.91	91.21	95.96	100.00	94.70	90.38	90.93	92.75	91.63	93.58	92.19
**17**	90.52	91.07	92.47	93.31	93.72	92.33	88.84	92.33	91.07	91.63	90.79	91.21	89.96	100.00	95.96	95.26	90.79	91.63	91.49	92.61	94.00	91.91
**18**	90.93	91.21	92.33	93.03	93.72	92.19	88.98	92.61	91.49	90.93	91.63	91.91	90.10	95.26	94.70	100.00	90.79	91.21	91.21	92.75	93.31	92.33
**19**	89.96	90.66	91.21	90.52	91.21	90.66	88.56	90.10	90.66	90.52	90.24	91.35	90.10	92.61	91.63	92.75	90.66	91.21	91.35	100.00	91.91	90.66
**20**	89.68	88.28	89.12	89.96	88.84	89.40	100.00	89.54	89.96	89.68	88.84	89.40	88.84	88.84	87.87	88.98	89.54	89.26	88.70	88.56	89.12	88.70
**21**	91.35	91.49	91.35	91.21	91.49	91.49	88.70	92.47	90.38	92.33	91.35	91.35	92.75	91.49	92.75	91.21	90.52	90.38	100.00	91.35	91.21	91.77
**22**	90.38	90.79	91.07	92.33	92.47	93.17	89.40	92.33	94.98	94.28	97.21	100.00	91.63	91.21	91.91	91.91	90.52	89.82	91.35	91.35	91.35	90.66

1: *E. asburiae*, 2: *E. bugandensis*, 3: *E. cancerogenus*, 4*: E. chengduensis*, 5: *E. chuandaensis*, 6*: E. cloacae*, 7: E. *dissolvens*, 8: *E. hoffmannii*, 9: *E. hormaechei,* 10: *E. huaxiensis*, 11: *E. kobei,* 12: *E. ludwigii*, 13: *E. mori*, 14: *E. oligotrophica*, 15: *E. quasihormaechei*, 16: *E. quasimori*, 17: *E. quasiroggenkampii*, 18: *E. roggenkampii,* 19: *E. sichuanensis*, 20: *E. soli*, 21: *E. wuhouensis*, 22: *E. xiangfangensis*

SeqScape Software 4 – Library Search Report

Generated at: 22 Jul 2021 at 17:25:18 CEST

Library

Library	Alleles #	Length	Haplotype	Polymorphic %	Creation Date	Modification Date	Comments
ECC_DNAJ_Type Strains	22	714	no	26.0	08 Jul 2021 at 16:58:32 CEST	08 Jul 2021 at 17:40:07 CEST	

Specimen	Library Sequence	Score	Mismatches in Constant Pos	Mismatches in Polymorphic Pos	Total Mismatches
S13	Enterobacter_bugandensis	950.0	0	0	0
S13	Enterobacter_chuandensis	608.0	0	38	38
S13	Enterobacter_mori	509.0	0	49	49
S13	Enterobacter_asburiae	509.0	0	49	49
S13	Enterobacter_roggenkampii	509.0	0	49	49
S13	Enterobacter_quasiroggenkampii	500.0	0	50	50
S13	Enterobacter_chengduensis	500.0	0	50	50
S13	Enterobacter_quasimori	500.0	0	50	50
S13	Enterobacter_quasihormaechei	473.0	0	53	53
S13	Enterobacter_hormaechei	464.0	0	54	54
